# The next-generation polyene EL219 is efficacious in an experimental model of central nervous system coccidioidomycosis caused by *Coccidioides immitis*

**DOI:** 10.1128/aac.01469-25

**Published:** 2026-01-26

**Authors:** Nathan P. Wiederhold, Laura K. Najvar, Rosie Jaramillo, Marcos Olivo, Thomas F. Patterson

**Affiliations:** 1 Fungus Testing Laboratory, Department of Pathology and Laboratory Medicine, University of Texas Health Science Center at San Antonio 14742, San Antonio, Texas, USA; 2 Division of Infectious Diseases, Department of Medicine, University of Texas Health Science Center at San Antonio 14742, San Antonio, Texas, USA; University Children's Hospital Münster, Münster, Germany

**Keywords:** EL219, *Coccidioides*species, coccidioidomycosis

## Abstract

We evaluated the *in vivo* activity of EL219 against central nervous system coccidioidomycosis. Mice were inoculated intracranially with arthroconidia of *Coccidioides immitis*, and treatment with EL219 (5, 10, or 20 mg/kg QD by intraperitoneal injection) or fluconazole (25 mg/kg orally BID) began 2 days later. Each dose of EL219 and fluconazole significantly improved survival. Brain fungal burden was also reduced compared to vehicle control. Further studies of EL219 against coccidioidomycosis are warranted.

## INTRODUCTION

Coccidioidomycosis is an invasive mycosis caused by the dimorphic *Coccidioides* spp., *Coccidioides immitis* and *Coccidioides posadasii*, which are endemic to warm, arid regions in the western hemisphere. This includes areas of Arizona, California, New Mexico, Texas, Utah, and Washington State in the United States (1, 2), although this endemic area appears to be increasing (3, 4). *Coccidioides* spp. are primary pathogens capable of causing disease in otherwise healthy, non-immunocompromised individuals who are exposed to these fungi (2), and in most individuals, an asymptomatic-to-mild respiratory infection occurs following inhalation of *Coccidioides* arthroconidia into the lungs. Disseminated disease, including central nervous system (CNS) infections, can occur in between 1% and 3% of individuals (5). In these cases, fluconazole is often prescribed because of its safety profile and good CNS penetration (5). Since relapses occur when fluconazole is stopped, therapy must be continued indefinitely for CNS disease (6). There is also concern for reduced *in vitro* susceptibility and *in vitro* resistance to fluconazole (e.g., MICs ≥ 32 μg/mL) in *Coccidioides* clinical isolates (7).

EL219 (previously SF001 and AM-2-19_DP2K) is a novel, next-generation polyene that is being developed for the treatment of various mycoses (8). It was rationally designed to reduce systemic toxicity, including nephrotoxicity, while maintaining the potency and broad-spectrum activity associated with other polyenes. Broad spectrum activity has been reported *in vitro*, including against both *C. immitis* and *C. posadasii*, with MICs ranging from 0.06 to 0.125 μg/mL against a limited number of strains (six per species) (8–10). *In vivo* activity has also been demonstrated in different animal models of invasive mycoses (8, 10, 11). In this proof-of-concept study, we evaluated the *in vivo* activity of EL219 against CNS coccidioidomycosis caused by *C. immitis*.

To establish CNS infection, immunocompetent male Institute of Cancer Research ICR mice (Envigo, Indianapolis, IN, USA) were anesthetized with isoflurane and underwent intracranial inoculation with a clinical *C. immitis* strain (UTHSCSA DI17-143; EL219 and fluconazole MICs of 0.06 and 8 μg/mL, respectively, as determined by Clinical and Laboratory Standard Institute broth dilution methods) as previously described (12–14). Briefly, each mouse was inoculated with a 0.06 mL volume using a 27-gauge needle for an infecting inoculum of 53 to 65 arthroconidia/mouse. The needle was fastened to a tuberculin syringe with a cuff to prevent penetration of more than 1mm, and a midline puncture through the cranial vault approximately 6 mm posterior to the orbit was made, followed by injection of the arthroconidia. To allow CNS disease to develop, the start of therapy was delayed for 48 hours after inoculation. In the fungal burden arm, treatment continued for 7 days (days 2–8) and for 14 days (days 2–15) in the survival arm. Treatment groups (10 mice per group) consisted of vehicle control (dextrose 5% wt/vol water [D5W]), EL219 at doses of 5, 10, and 20 mg/kg QD by intraperitoneal injection, or fluconazole 25 mg/kg BID (total daily dose of 5 mg/kg) by oral gavage. The EL219 doses used are within the range studied by others in different experimental models of invasive fungal infections (8, 10, 11). The fluconazole dose is consistent with previous studies by our group where this agent has been used as a positive control in this model (12, 13). In the survival arm, treatment was stopped after the last dose on day 15, and mice were monitored off therapy until day 30 post-inoculation. Animals that appeared moribund by pre-specified criteria were humanely euthanized, and death was recorded as occurring the next day. In the fungal burden arm, brain fungal burden was measured on day nine post-inoculation (1 day after the last treatment dose), and in the survival arm, this was done either at the time of euthanasia or the predefined study endpoint (day 30). Brains were aseptically collected, weighed, and homogenized in sterile saline. Aliquots of the homogenates were then plated onto potato dextrose agar, and colony-forming units (CFUs) were enumerated following incubation at 37°C for 72 to 96 hours. Fungal burden was measured as CFUs per gram of tissue (CFU/g). If no CFUs were observed, the entire brain homogenate was plated and allowed to incubate at 37°C for confirmation. Survival was plotted by Kaplan-Meier analysis, and differences in median and percent survival to day 30 were analyzed by log-rank and Fisher’s exact test, respectively. Differences in CFU/g among the different groups were assessed by the Kruskal-Wallis test with Dunn’s correction for multiple comparisons. *P*-values of ≤ 0.05 were considered statistically significant.

**Fig 1:**
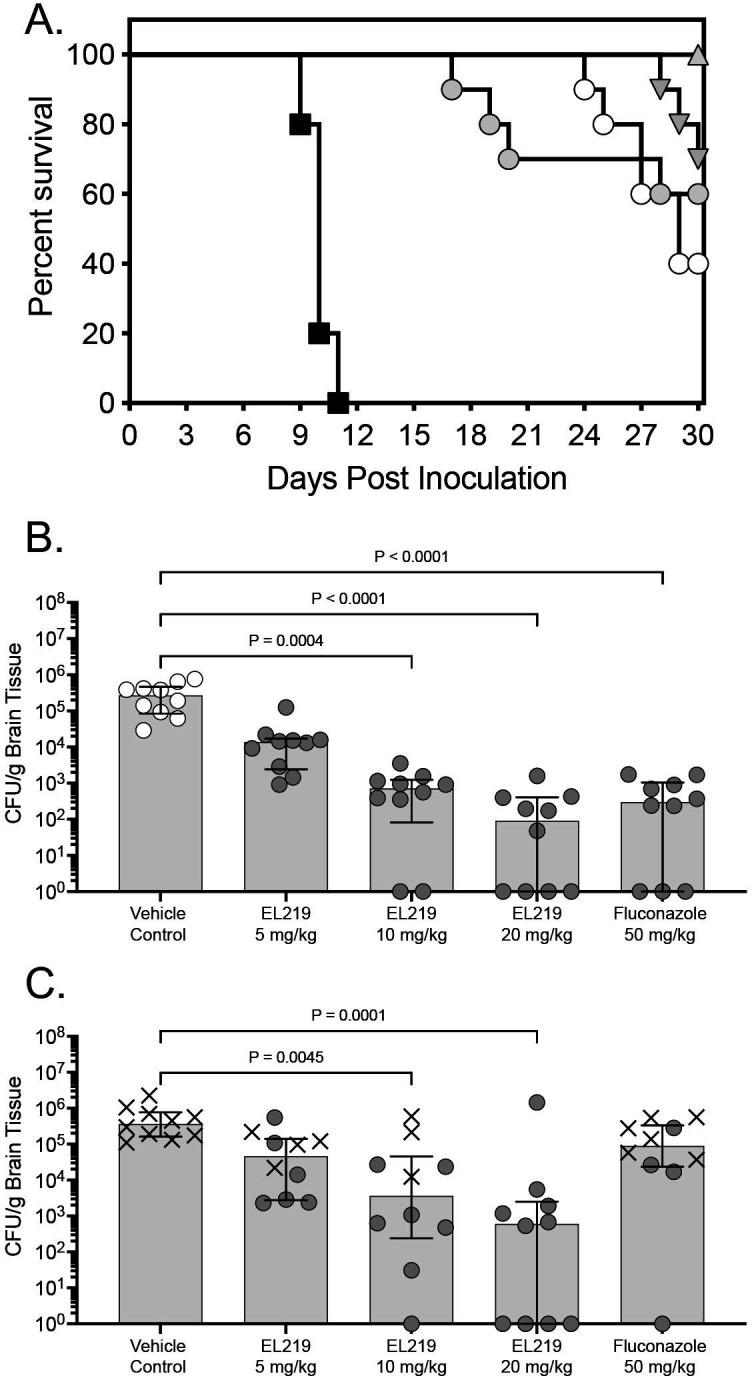
Survival curves in mice with CNS coccidioidomycosis and treated with EL219 or fluconazole (**A**), fungal burden on day 9 post-inoculation (**B**), or fungal burden in the survival arm (day 30 or as mice became moribund; **C**). Treatment was started 2 days post-inoculation and was continued for 7 days in the fungal burden arm or 14 days in the survival arm. In the survival arm, mice were then monitored off therapy until day 30. X denotes mice that were deemed moribund prior to the day 30 study endpoint in the survival arm (**C**). For fluconazole, the dose was 25 mg/kg BID for a total daily dose of 50 mg/kg.

EL219 improved survival in this murine model of CNS coccidioidomycosis at each dose level evaluated. Both median survival (>30 days) and percent survival (range 60% to 100%) at day 30 post-inoculation were significantly improved compared to vehicle control (10 days and 0%; *P* ≤ 0.01 for all comparisons) (Fig. 1A). Similar results were also observed with fluconazole (29 days and 40%, respectively), although percent survival at day 30 did not reach statistical significance versus vehicle control (*P* = 0.09). In the fungal burden arm, treatment with EL219 also significantly reduced brain fungal burden on day 9 post-inoculation with the 10 and 20 mg/kg dosage groups (median range 1.96 to 2.85 log_10_ CFU/g) compared to vehicle control (5.43 log_10_ CFU/g; *P* ≤ 0.0004 for each comparison; Fig. 1B). Similar results were also observed with fluconazole (2.47 log_10_ CFU/g; *P* < 0.0001 vs control). In the survival arm, fungal burden was also significantly reduced in both the EL219 10 and 20 mg/kg dosage groups (median range 2.78 to 3.56 log_10_ CFU/g) compared to vehicle control (5.56 log_10_ CFU/g; *P* ≤ 0.0045 for each comparison; Fig. 1C). In contrast, fungal burden in the fluconazole group (5.42 log_10_ CFU/g) was similar to that of vehicle control. In all groups, rates of survival were significantly greater in mice in which fungal burden was below 4.0 log_10_ CFU/g (18 of 27; 66.7%) compared to those in which it was higher than this threshold (0 of 23; 0%; *P* < 0.0001). This was especially evident in the EL219 20 mg/kg group in which 9 of 10 mice had fungal burden less than 4.0 log_10_ CFU/g and survival was 100% at day 30. The fluconazole results in both the survival and fungal burden arms were consistent with those from previous studies by our group (12, 13, 15), indicating that the model performed as intended.

The results of this study add to the literature regarding the *in vivo* activity of EL219 against invasive mycoses. In initial studies, EL219 was shown to be effective against infections caused by *Candida albicans*, *Candida glabrata* (*Nakaseomyces glabratus*), *Aspergillus fumigatus*, *Aspergillus terreus*, and *Rhizopus delemar* (8). More recently, it has been reported to be efficacious in a murine model of fusariosis, resulting in improvements in survival and reductions in kidney and brain fungal burden, which was corroborated by histopathological findings (11). In a murine model of invasive pulmonary aspergillosis, both the area under the curve/MIC and *C*_max_/MIC pharmacokinetic/pharmacodynamic (PK/PD) parameters were strongly associated with efficacy with a steep dose-response curve (10). Notably, the EL219 doses that improved survival and reduced fungal burden in this murine model of CNS coccidioidomycosis are similar to those that were efficacious in other experimental models of invasive fungal infections (8, 10, 11). Overall, the results of this study and those of others suggest that further studies of EL219 against coccidioidomycosis and invasive mycoses are warranted, including comparisons with other antifungals at clinically relevant exposures.
