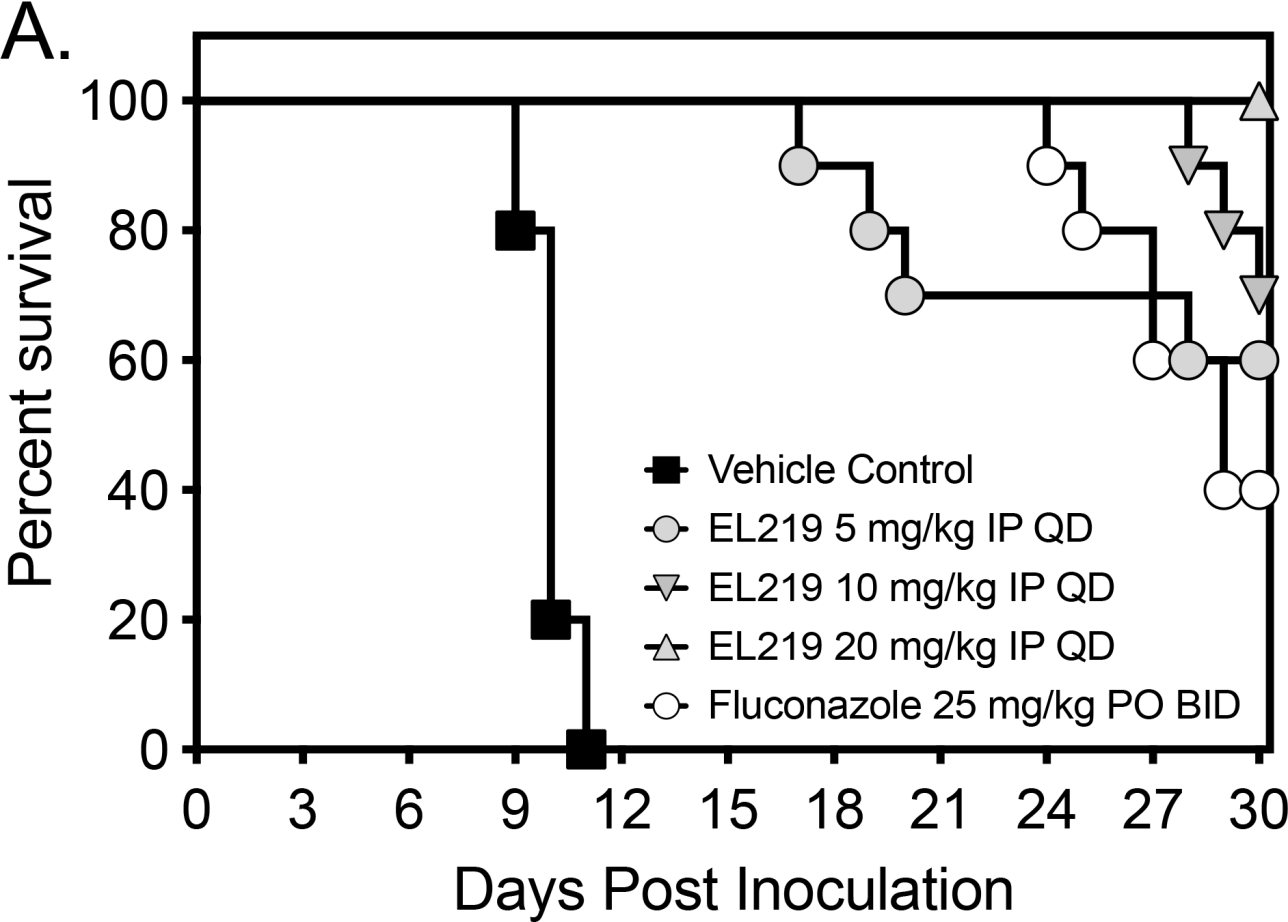# Erratum for Wiederhold et al., “The next-generation polyene EL219 is efficacious in an experimental model of central nervous system coccidioidomycosis caused by *Coccidioides immitis*”

**DOI:** 10.1128/aac.00292-26

**Published:** 2026-06-04

**Authors:** Nathan P. Wiederhold, Laura K. Najvar, Rosie Jaramillo, Marcos Olivo, Thomas F. Patterson

## ERRATUM

Volume 70, no. 3, e01469-25, 2026, https://doi.org/10.1128/aac.01469-25. Figure 1: In panel A, the legend showing which symbols correspond to which treatment groups was inadvertently left off. The data presented are correct.

**Fig 1:**